# The reproductive success of Simmental bovine after sex-sorting under various incubation and centrifugation protocols

**DOI:** 10.14202/vetworld.2023.631-637

**Published:** 2023-03-26

**Authors:** Langgeng Priyanto, Herdis Herdis, Santoso Santoso, Rahma Isartina Anwar, Tri Puji Priyatno, Pradita Iustitia Sitaresmi, Faiz Azhari, Muhammad Gunawan, Oktora Dwi Putranti

**Affiliations:** 1 Department of Animal Science, Faculty of Agriculture, Sriwijaya University, South Sumatra, 30862, Indonesia; 2 Research Center for Animal Husbandry, National Research and Innovation Agency, Cibinong Science Center, Jalan Raya Jakarta-Bogor, Bogor, 16915, Indonesia; 3 Department of Animal Husbandry, Faculty of Agriculture, Animal Husbandry of Universitas Khairun, Ternate, North Maluku, Indonesia

**Keywords:** bovine serum albumin, centrifugated, conception rate, incubation, sexing, sperm

## Abstract

**Background and Aim:**

To enhance the reproductive potential and increase productivity and population of cows, spermatozoa sex-sorting technology is required. This study aimed to examine the effect of sexing sperm, separated using a bovine serum albumin (BSA) column with varying incubation durations and centrifugation methods, for successful artificial insemination.

**Materials and Methods:**

Six Simmental bulls and 30 cows (n = 30) as the recipients were selected for this study at Balai Pembibitaan Hijauan Pakan Ternak Sembawa Indonesia. The study parameters included sperm motility, viability, plasma membrane integrity, and conception rate (CR). The experiment was divided into three protocols to find out differences in some parameters: (1) BSA incubation time effect (P) with P1 (40 min), P2 (50 min), and P3 (60 min); (2) freezing time effect with before freezing and after-thawing treatments; and (3) CR determined by measuring the proportion of pregnant cows following insemination with non-sexed, X-bearing, and Y-bearing sperms without centrifugation (n = 15) (A0, A1, and A2) and with centrifugation (n = 15) (B0, B1, and B2) in the acquired data, which were counted using the Statistical Package for the Social Sciences version 21 program. Analysis of variance was utilized to evaluate all treatments at various levels.

**Results:**

The results demonstrated that centrifugation time influenced all sperm quality metrics for sperm containing X and Y (p < 0.05). The non-return rate (NRR) of non-sexed frozen semen, both centrifuged (A0) and not centrifuged (B0), was more significant than frozen semen produced by sexing X and Y spermatozoa. The NRR indicated a value of 80% based on the number of lactating cows.

**Conclusion:**

Bovine serum albumin incubation and centrifugation protocols influenced and decreased all sperm quality indicators throughout the sexing procedure and could still be used as a sexing protocol. Furthermore, regarding NRR and service per conception, non-sexual treatment is superior to sexing treatment.

## Introduction

Progeny selection of a particular sex is one of the most effective methods for increasing the genetic advancement and profitability of cattle farms [1]. Bull calves are preferred for meat production, while cow calves are preferred by the dairy industry [2], and sexed semen is crucial for producing offspring of the desired gender [3, 4]. Therefore, the gender balance of offspring arising from natural mating (the chance of male calves is fixed at a ratio of 51:49, which is one of the few genetic features that breeding programs cannot effectively control or change) or artificial breeding programs can be genetically controlled [5].

The presence of either X- or Y-chromosome-bearing sperm in the sexed semen enabled the creation of offspring of the selected sex [6]. Various approaches have been used, such as flow cytometry, albumin sedimentation, and Percoll density-gradient centrifugation, to differentiate chromosome X sperm and Y sperm based on their DNA content differential ranges (3.7%–4.2%), depending on the breed [7]. One of the simple and many used methods was the bovine serum albumin (BSA) gradient method. This method does not damage the acrosomal integrity of sperm or sexed sperm yield, which is one of the reasons why it is preferred. Bovine serum albumin column methods have a conception rate (CR) similar to that of conventional semen of more than 85% [8] or use egg white albumin [9]. This technique is expected to prevent a decline in the quality of spermatozoa after the sexing process, because the BSA gradient method does not excessively manipulate spermatozoa [10].

Although sexing sperm is one of the most intensively researched technologies and significant progress has been achieved in optimizing it over the past three decades, CR, when employing sex-sorted sperm, is still below expectations. Furthermore, proving the success of the conclusions of this study in practical applications is rare.

This study aimed to verify the spermatozoa carrying the X- and Y-chromosomes that have been separated using a 5%–10% concentration BSA column at various incubation times and the effect of the centrifugation process on the quality of the semen also produced to calculate the percentage success in the field of male and female births using the artificial insemination method affected by previous treatment.

## Materials and Methods

### Ethical approval

All animal procedures were performed according to the guidelines for the care and use of experimental animals of the National Research and Innovation Agency (BRIN) Indonesia with the number 065/KE.02/SK/2022.

### Study period and location

The study was conducted from January to September 2022 at the Balai Pembibitaan Hijauan Pakan Ternak (BPHPT) in Sembawa, Banyuasin, South Sumatra, Indonesia,

### Semen sample collection

Samples of sperm from six domesticated Simmental bulls aged 4–5 years (measured 380–450 kg BW) were collected and stored separately in a refrigerator (4°C) without any diluent supplementation. The bulls were fed with a combination of forages (10% BW) and concentrate (1% BW) twice per day and water was provided as *ad libitum*. All bull in this research as the hustler in BPHPT and as the semen producer/donor in Sumatera area. Laboratory for Animal Reproduction and Health of BPHPT Sembawa Indonesia has also enacted laws and regulations governing animal experimentation. Samples of sperm were obtained using an artificial vagina collection. The only good quality sperm samples used in the experiment which had a sperm concentration of >800 × 10^6^ cells/mL and total motility of <60%.

### Sexing sperm using BSA column

Four-cylinder tubes were used to prepare BSA column, which was then inflated to the bottom with a 10% concentration and the top with a 5% concentration. Each container was kept at 37°C and 27°C. Then, fresh sperm was diluted with tris egg yolk medium; 1 mL sample was placed in a tube containing 5% and 10% BSA columns, according to the treatment. The final sperm concentration was 200 million/mL. After 30 min, each tube of sperm was placed in a tube rack and stored in a water bath at 37°C and laminar cabinets at room temperature (27°C).

Each BSA column was divided into three groups, and each sample was incubated for 40, 50, and 60 min (P1, P2, and P3). It was projected that the upper BSA column with a concentration of 5% would contain X-chromosome sperm, and the lower column with a concentration of 10% would contain Y-chromosome sperm. Diluted sperm was packaged in a mini straw and equilibrated at 5°C for 4 h in the refrigerator. Then some straws were frozen in a box containing liquid nitrogen for 10–15 min before being stored in a nitrogen container. The others would direct sperm quality testing.

### Parameters of sperm quality

The study’s parameters were sperm motility, viability, intact plasma membrane, and CR. The study was divided into three groups: Bovine serum albumin incubation time (P) with P1 (40’), P2 (50’), and P3 (60’) min incubation in BSA, and freezing time with before freezing (BF) and after-thawing (AT) treatments. The data obtained in this study, such as motility, viability, abnormalities, intact plasma membrane, and conception rate, were tallied in the IBM SPSS Statistics for Macintosh, Version 21.0 (IBM Corp., NY, USA). An analysis of variance was used to examine all treatments.

### Semen evaluation

The data observed were concentration, motility, viability abnormalities, and plasma membrane integrity/HOST of spermatozoa before and after freezing. The sperm motility was followed by putting and homogenizing 10 mL of diluent mixed with NaCl (1:4) and then placing it on the microscope (Olympus CH 20, Boston, MA, USA). Slide viewed was taken at ten fields with a magnification of 100 × 400; scores were given in the range 0–100% with a 5% scale. The eosin staining procedure was used for sperm viability. A total of 200 spermatozoa were counted per sample using a light microscope (Olympus CH 20) to differentiate the reacted and non-reacted spermatozoa. The dead sperm with damaged acrosomes emitted a robust red color, whereas non-reacted with live sperm emitted light pink or no shade. Based on the coiled and swelled tails, the hypo-osmotic swelling test was utilized to determine the functional integrity of the sperm membrane. This was accomplished by incubating 0.1 mL of sperm with 1 mL of a 150 M hypo-osmotic solution at 37°C for 30 m. After incubation, 0.2 mL of the solution was distributed on a warm microscope slide using a cover slip. One thousand times magnification was used to examine 200 spermatozoa under bright-field microscopy. Abnormality in sperms was recorded and plasma membrane damage would be inflated or had curled tails [11].

### Non-return rate (NRR)

Conception rate was obtained to measure NRR by calculating the percentage of pregnant cows after insemination using non-sexed sperm, X-bearing sperm, and Y-bearing sperm without centrifugation (n = 15) (A0, A1, and A2) and non-sexed sperm, X-bearing sperm, and Y-bearing sperm with centrifugation (n = 15) (B0, B1, and B2) in the first insemination of the total number of cattle inseminated. The data collected were calculated using the formula [12]:


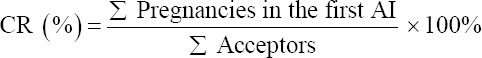


Where,

∑ Acceptors: Artificially inseminated cows

∑ Pregnancies in the first AI: Total cows considered pregnant

### Service per conception (S/C)

Service per conception was obtained by determining the number of straws used and the number of pregnant females. The data collected were calculated using the formula [12]:


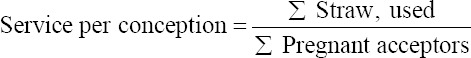


Where,

∑ Pregnant acceptors: Total pregnant females

∑ Straw used: The number of staws used until the cattle are pregnant

## Results and Discussion

### The sperm quality of fresh semen of Simmental Cattle

The successful use of sexed sperm in bovines has been documented; the most common application of sexed sperm is for the sex preselection of bulls to achieve an adequate number of national beef cattle. Utilizing sexed sperm is an effective method for producing offspring of a particular gender [2, 12]. Several separation methods, such as the use of an albumin column with BSA, have been employed. Bovine serum albumin (serum albumin protein) protects sperm by protecting the plasma membrane from free radical damage. An accurate combination of BSA concentrations maintains optimal sperm quality during sexing [13]. Table-1 shows that the average fresh semen for each cattle was 3.5 ± 0.707 mL, which is still in normal conditions (2–19 mL per ejaculation) [14]. In addition, all parameters appeared normal, and fresh semen samples met the standard requirements for the semen sexing process in further experiments [3]. The motility of fresh semen to be processed into frozen semen should be at least 70% for a bull. If the motility is <70%, it can still be used if the recovery rate is at least 50% (BSN, 2017). Production of frozen sexed semen using 5% and 10% BSA columns can only be performed if the motility percentage value is 60% to anticipate a drastic decrease in sperm quality due to the incubation treatment for 40–60 min longer than the usual freezing process [8]. In addition, the sperm was 1750 ± 100 × 10^6^ cells/mL. This concentration was considered typical. According to previous research, the standard concentration of bull sperm is 800–2000 × 10^6^ cells/mL. This standard is consistent with our analysis; consequently, the sperm used in this study could be processed further [15].

**Table-1:** Macroscopic quality of Simmental bull fresh semen.

Parameter	Values
Volume (mL)	3.5 ± 0.71
Color	Creamy
Odor	Typical
Ph	6.85 ± 0.06
Consistency	Medium
Concentration (×10^6^/mL)	1750 ± 100
Motility mass	++
Motility	82.5 ± 5.00
Viability	89.84 ± 8.00

++ (positive 2)=Thick mass waves but slow-moving

### Effect of BSA incubation time on sexing spermatozoa on motility and viability of spermatozoa X and Y of Simmental cattle

One of the sperm sexing methods is the BSA gradient method. This procedure is expected to prevent a deterioration in the quality of spermatozoa following sexing, as the BSA gradient method is not thought to alter spermatozoa excessively. Spermatozoa sexing is often accomplished by separating the X- and Y-chromo­somes based on differences in deoxyribonucleic acid (DNA) content, physical traits, macro proteins, and weight and motility of spermatozoa [10]. A previous study reported that 5% BSA had a pH of 7.43, density of 1.0547 g/mL, and viscosity of 0.8648 cP, whereas 10% BSA had a pH of 7.40, density of 1.0661 g/mL, and viscosity of 1.0378 cP. This characteristic of BSA is one of the reasons for sexing semen separation [3]. The neutral pH of BSA places the spermatozoa in a comfortable condition through the albumin column. This is because sperm do not change the internal pH.

**Figure-1:**
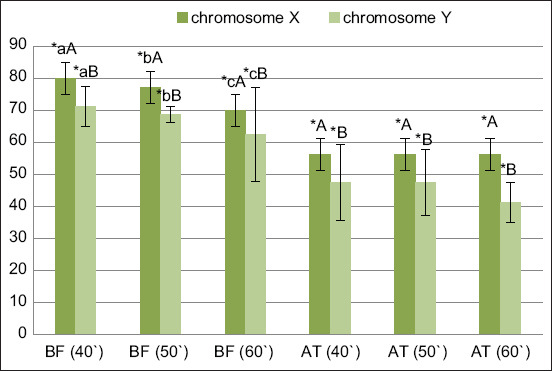
Effect of time incubation of bovine serum albumin treatment on sperm motility and the effect of cryopreservation (before freezing [BF]). Mean sperm motility BF; and (after-thawing [AT]) mean P/AI for each treatment within bull. Incubation time; 40, 50, and 60 min. Data reported as least square means ± standard deviation. ABC showed a significantly differ (p < 0.05) in the effect of incubation time; the data showed time incubation affected to change the motility either in X or Y sperm chromosome except in AT condition. AB showed a significantly differ (p < 0.05) by sorting X and Y sperm chromosome in each treatment. **Showed significantly differ in cryopreservation treatment before and AT.

**Figure-2:**
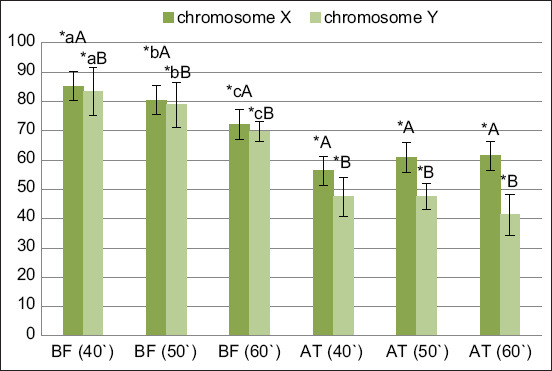
Effect of time incubation of bovine serum albumin treatment on sperm viability and the effect of cryopreservation (before freezing [BT]). Mean sperm viability BT; and (after-thawing [AT]) mean P/AI for each treatment within bull. Incubation time; 40, 50, and 60 min. Data reported as least square means ± standard deviation. ABC showed a significantly differ (p < 0.05) in the effect of incubation time; the data showed time incubation affected to change the motility either in X or Y sperm chromosome except in AT condition. AB showed a significantly differ (p < 0.05) by sorting X and Y sperm chromosome in each treatment. **Showed significantly differ in cryopreservation treatment before and AT.

The quality of spermatozoa post-incubation on the BSA column is shown in Table-2 and the next protocol was the freezing method. Based on the study data, the average BF or fresh semen quality of X and Y sperm was the highest (p *<* 0.05) in P1 (40 min incubation time), with 80% and 85.3% in X sperm and 71.25% and 83.84% in Y sperm motility and viability, respectively, and the lowest values were found in P3 (60 min incubation time) (Table-2, Figures-1 and 2). However, no significant effect of the BSA incubation time was observed after semen thawing. This result was similar to that of BSA media sexing semen in local Indonesian rams [16], which also showed that incubation time significantly affects the viability of X and Y sperms. The longer the incubation period, the greater the accumulation of lactic acid from cell metabolic activities, which results in an acidic environment and the generation of reactive oxygen species that promote lipid peroxidation through oxidation processes that bind to cell membranes. These conditions reduce sperm motility or viability [16].

**Table-2:** Effect of time BSA incubation media on sexing semen procedure in motility and viability semen before and after freezing procedures.

Parameter	X-bearing sperm (BSA 5%)			Y-bearing sperm (BSA 10%)		
	
	P1 (40`)	P2 (50`)	P3 (60`)	P1 (40`)	P2 (50`)	P3 (60`)
Motility (%)						
Before freezing	80.00*^aA^ ± 8.17	77.5*^bA^ ± 5.00	70*^cA^ ± 14.14	71.25*^aB^ ± 6.29	68.75*^bB^ ± 2.50	62.5*^cB^ ± 14.72
After-thawing	56.25*^A^ ± 2.5	56.25*^A^ ± 11.09	56.25*^A^ ± 11.91	47.5*^B^ ± 11.90	47.5*^B^ ± 10.40	41.25*^B^ ± 6.29
Viability (%)						
Before freezing	85.30*^aA^ ± 9.37	80.40*^bA^ 6.81	72.11*^cA^ ± 12.07	83.84*^aB^ ± 8.26	78.82*^bB^ ± 7.53	69.61*^cB^ ± 3.46
After-thawing	56.33*^A^ ± 6.18	60.87*^A^ ± 9.56	61.42*^A^ ± 6.91	48.71*^B^ ± 6.62	55.18*^B^ ± 4.38	44.47*^B^ ± 6.88

* Total means with different superscripts within a row differs significantly (p < 0.05), freezing treatment effect. ^abc^Total means with different superscripts within a column differs significantly (p < 0.05), incubation time treatment effect. ^AB^Total means with different superscripts within a group column differs significantly (p < 0.05), chromosome factor after the incubation. BSA=Bovine serum albumin

Moreover, X sperms showed longer viability than Y sperms in long-term incubation. X-sperm may save more energy (shown with lower motility in X sperm than Y sperm) while keeping the membrane more intact than Y sperm due to their wider heads and slower movement [17]. Ligand activation of toll-like receptors, 7/8 in X-encoded sperm, suppresses motility without affecting fertilization [18]. Other reasons described in the human sperm findings state that the viability of mammalian Y spermatozoa is lower than that of X spermatozoa due to the increased expression of apoptotic proteins in live Y cells [19]. In addition, we assumed that the greater the concentration of BSA, the greater the viscosity and density; therefore, Y sperms in the lower layer encountered greater friction. This frictional strain causes severe membrane damage to the bottom layer of the sperm.

Due to the cryopreservation process, all parameters of sperm quality AT revealed a significant (p *<* 0.05) reduction in motility and viability but not significant in each incubation time treatment. This is similar to a previous research that stated that the freezing-thawing mechanism targets sperm DNA and protaminesolysis and leads to decreased quality parameters after the process [20]. According to a previous study, freeze-thaw cycles lead to increased DNA breakage. In this study, chromatin dispersion (the halo surrounding the nucleus) and the loss of protamine in the abnormal sperm cell population were indicative of DNA fragmentation (deprotamination). DNA fragmentation in the sperm cells is associated with elevated levels of deprotamination, which increases the risk of infertility [21]. The insufficient data on viability AT can also be attributed to the fact that this stage did not include a centrifugation treatment. In those samples, dead sperm cells were still counted in the viability calculation after the BSA treatment, which requires more than 30 min, because the purpose of centrifugation in sexing spermatozoa is to separate live and dead spermatozoa from other hazardous substances. The data found in the after-thawing condition were different from those before the freezing event; however, the differences were not significant, as longer incubation times resulted in higher viability, except for P3 in the Y-chromosome-bearing sperm. Incubation is an important stage in sperm cryopreservation because it concentrates the live sperm population such that it can be re-diluted with freezing extenders to prevent cell viability AT.

### Conception rates of Spermatozoa X-Y Simmental Cattle on BSA sexing media with or without centrifugation

The conception rates after incubation on the BSA column with or without centrifugation are shown in Table-3. Based on the results of the study, the NRR values of frozen non-sexed semen, both centrifuged (A0) and un-centrifuged (B0), were greater than those of frozen semen produced by sexing X and Y spermatozoa. Non-return rate (both A_0_ and B_0_) showed a value of 80%, with the number of cows in heat again after AI being one heat female.

**Table-3:** Effect time of centrifugation procedures after sperm separating using BSA procedure in conception rates parameters.

Parameter	Without centrifugated			With centrifugated 8 min		
	
	Non-sexed A_0_	X-bearing sperm N: 5 A_1_	Y-bearing N: 5 A_2_	Non-sexed B_0_	X-bearing sperm N: 5 B_1_	Y-bearing N: 5 B_2_
NRR						
NRR 1 (30 days)						
Non-heat	4	3	3	4	4	3
% animals	80	60	60	80	80	60
NRR 2 (40 days)						
Non-heat	4	2	3	4	3	3
% animals	80	40	60	80	60	60
NRR 3 (60 days)						
Non-heat	4	2	3	4	3	2
% animals	80	40	60	80	60	40
C/R						
Animals	4	2	3	4	3	2
% animals	80	40	60	80	60	40
S/C	1.25	2.5	1.66	1.25	1.6	2

S/C=Service per conception, C/R=Critically endangered, BSA=Bovine serum albumin, NRR=Non-return rate

Non-return rate (A_1_) decreased to 40%, NRR3 from 60% for NRR1, and the number of cows came in heat again after AI being two acceptors at the end of the examination. The NRR value for (A_2_) was 60%, with two cows in heat again after AI being two females. The NRR for (B_1_) decreased from 60% for NRR3 to 80% for NRR1, with the number of cows in heat again after AI being the two acceptors at the end of the examination. The NRR value for (B_2_) was 40%, with three cows in heat again after AI being three females. The NRR for A_0_ and A_2_ is in the excellent category (>50%), and the NRR for (A_1_) in this study is in the unsatisfactory category (<50%). Despite this, the NRR for B_0_ and B_1_ is in the excellent category (>50%), and the NRR for (B_2_) in this study was in the unsatisfactory category (<50%). Meanwhile, a good NRR value is 79.53% [22]. The interesting data in this study was the sample which centrifuged had a higher NRR than the sample without centrifuged. We assumed that this was because centrifugation aids in the elimination of seminal plasma, concentrates spermatozoa for redilution using cryopreservation extenders, and improves the quality of the sperm itself.

**Figure-3:**
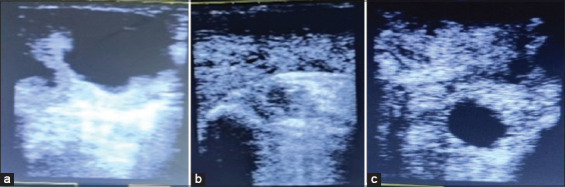
Ultrasonography images monitoring pregnancy rate (Source: Personal collection, 2022). (a) Day 6, (b) day 21, and (c) day 30.

In this study, each day, the animals were undergone an ultrasound examination to monitor the condition of the uterus and as an attempt to detect pregnancy, especially in early pregnancy, which is presented in Figure-3. Based on the CR values, the AI results of AI using non-sexed semen were higher than those obtained using sexed semen. The CR values of non-sexed spermatozoa (A_0_), sexed X-spermatozoa (A_1_), and sexed Y spermatozoa (A_2_) were 80%, 40%, and 60%, respectively, on un-centrifuged semen. Meanwhile, the CR values of non-sexed spermatozoa (B_0_), sexed X-spermatozoa (B_1_), and sexed Y spermatozoa (B_2_) were 80%, 60%, and 40%, respectively, on centrifugated semen. In this study, the CR values of (A_0_ and B_0_) and (A_2_ and B_1_) were better and in the excellent category than those of (A_1_ and B_2_), which were still considered unsatisfactory. Boro *et al*. [23] stated that the conception rate using sexing semen reached 45%.

Meanwhile, the standard CR in cows is 60%–70%. The low CR value of sexed sperm results from their low motility of sexed sperm following the sexing procedure, and a time requirement of more than 30 min for sexed sperm has many adverse effects on sperm cells. Sexing techniques reduce sperm motility, viability, and fertilization capacity. This phenomenon is associated with the energy source in the head of sexed spermatozoa; consequently, during the separation or sexing process, many sexed spermatozoa die on the way, or the number of spermatozoa decreases because the separated spermatozoa undergo a treatment that requires a great deal of energy to maintain their physiological conditions [14].

The lowest S/C value was observed in the non-sexed treatment semen (A_0_ and B_0_), with 1.25 still significantly lower than that of the sexed semen (Table-3). When the S/C ratio was low, the fertility value of the cows was high and when the S/C ratio was high, the fertility value of the cows was low. As per a previous study, the normal range of S/C values is 1.6 and 2.0, where the S/C values for (A_0_ and B_0_) are in an outstanding category, even though the sex treatment was still in the normal category [22]. As evidenced by the NRR1 and NRR2 data, centrifugation was superior to non-centrifugation in the centrifuged sample compared to non-centrifuged selection. Moreover, additional research is required to determine the optimal spin effect (*g* force variable from 300–10,000 rpm) and spin-time effect.

Other data indicate that the X-chromosome has higher parameters than sperm with high-quality Y-chromosomes, due to the energy-saving factor during the separation process with BSA. Therefore, suggestions can be made regarding alternative media that can separate sperm more quickly in future research, as well as the *in situ* hybridization method, which will aid in sexing success. Furthermore, we suggest finding a preservation agent to prevent severe damage from using similar methods, such as an antioxidant agent, in future research.

## Conclusion

Incubation time influenced all sperm quality parameters in the BSA method for sexing sperm. In terms of sperm quality, in general, the NRR and CR of frozen non-sexed sperm with the shortest incubation time (40 min) indicated superior sperm quality. The data also revealed that sperm containing an X-chromosome and centrifuged semen performed better in terms of sperm quality measures and post-insemination data.

## Authors’ Contributions

PIS, LP, and HH: Conducted the literature search and drafted the manuscript. ODP, RIA, FZ, and MG: Conceived the study design, performed the fieldwork, administrated the study, and helped in drafting the manuscript. LP and PIS: Conducted data interpretation and edited the manuscript. LP, PIS, TPP, SS, and HH: Designed and supervised the study. PIS, SS, TPP, and LP: Performed the statistical analysis and reviewed the manuscript. HH, TPP, and SS: Supervised the study. All authors have read, reviewed, and approved the final manuscript.
